# AiZynthFinder: a fast, robust and flexible open-source software for retrosynthetic planning

**DOI:** 10.1186/s13321-020-00472-1

**Published:** 2020-11-17

**Authors:** Samuel Genheden, Amol Thakkar, Veronika Chadimová, Jean-Louis Reymond, Ola Engkvist, Esben Bjerrum

**Affiliations:** 1 Hit Discovery, Discovery Sciences, R&D, AstraZeneca Gothenburg, Mölndal, Sweden; 2 Department of Chemistry and Biochemistry, University of Bern, Freiestrasse 3, 3012 Bern, Switzerland

**Keywords:** Neural network, CASP, Retrosynthesis planning software, Monte Carlo tree-search, Retrosynthesis

## Abstract

We present the open-source AiZynthFinder software that can be readily used in retrosynthetic planning. The algorithm is based on a Monte Carlo tree search that recursively breaks down a molecule to purchasable precursors. The tree search is guided by an artificial neural network policy that suggests possible precursors by utilizing a library of known reaction templates. The software is fast and can typically find a solution in less than 10 s and perform a complete search in less than 1 min. Moreover, the development of the code was guided by a range of software engineering principles such as automatic testing, system design and continuous integration leading to robust software with high maintainability. Finally, the software is well documented to make it suitable for beginners. The software is available at http://www.github.com/MolecularAI/aizynthfinder.

## Introduction

Synthesis planning is the process by which a chemist or a computer determines how to synthesize a specific compound. This is typically carried out by retrosynthetic analysis where the desired compound is iteratively broken down into intermediates or smaller precursors until known or purchasable building blocks have been found. Such analysis was pioneered by Corey et al. and was traditionally carried out by hand or by using expert systems utilizing hand-encoded rules [1–3]. With the rise of deep learning, in the last decade, the field of retrosynthetic software tools has undergone a swift change. Now, sophisticated and automatic algorithms have the potential to provide retrosynthetic analysis with a broader application domain and with better accuracy [4–6].

Retrosynthesis planning algorithms can be divided into template-based and template-free approaches. In template-based approaches, reaction templates or rules that describe chemical transformations are manually encoded or derived from a database of known reactions, and subsequently applied to other compounds to create plausible reaction outcomes. Segler et al. showed that it was possible to train a neural network to prioritize templates, and subsequently use this as a policy to guide a Monte Carlo tree search algorithm that suggests synthetic pathways for a given compound [7, 8]. Template-free approaches, on the other hand, do not rely on such templates but typically treat the chemical reaction as a natural language problem, where one set of words (reactants) is transformed into another set of words (products) [9–11]. Other template-free methods are based on graph approaches [12, 13].

There are several tools available for retrosynthesis planning but to our knowledge only two are fully open source, i.e. the ASKCOS suite of programs from MIT [14] and LillyMol from Eli Lilly and Company [15]. The tools Chemical AI [16] and IBM RXN [17] are free for registered users, but only the algorithm of the latter has been reported in the literature. Other tools [18–23] are closed commercial applications where the algorithm is partly undisclosed. This is partly a problem of data availability—most of the reaction databases or manually encoded rules are commercial and limits the way a free and open source software can use them. The same applies to the database of purchasable precursors that is used as a stop criterion in several programs. However, we believe that the scientific community would benefit from an open source implementation that provides algorithmic transparency and promotes reproducible research with a sustainable software. Therefore, we present the AiZynthFinder tool that can be used for retrosynthesis planning. An early version of this tool has been used previously to determine the influence of the reaction database on retrosynthesic predictions [24], but the code base has been re-engineered to make it more flexible, robust and maintainable. We provide a trained neural network policy as well as tools to make a database of purchasable precursors so that the tool can be used directly. In addition, we provide extensive documentation to lower the learning curve for new users. We envisage that by providing this tool free and open-source, other researchers can use it for benchmarking, contribute to a continuous development and use the tool for suggesting synthetic routes for novel compounds.

## Implementation

The AiZynthFinder software is written in Python 3 and is distributed on GitHub under the MIT license [25]. It is dependent on several freely available Python packages such as TensorFlow [26], RDKit [27] and NetworkX [28].

The central algorithm of the AiZynthFinder software has been described elsewhere [8, 24] and therefore, we only provide a brief outline here: The input is a molecule that will be broken down to purchasable precursors. The outcome will be a list of precursors that can be purchased or molecules that cannot be broken down by the algorithm. The software is based on a Monte Carlo tree search [29], where each node in the tree corresponds to a set of molecules that can or cannot be broken down further. At each iteration a leaf node is selected that is deemed to be the most promising to exploit further using upper confidence bound statistics [29]. A neural network policy is then used to shortlist reaction templates and prioritize which child to create by applying a reaction template to create the new precursors. This procedure is repeated until a terminal state has been reached, i.e., a precursor that is purchasable has been found, or the tree has reached a maximum depth. At this point the score of the leaf node is backpropagated up to the root of the tree (the input molecule), and the next iteration commences. The tree search is terminated either after a fixed number of iterations or a time-limit has passed. In comparison to the algorithm proposed by Segler et al. [8], the algorithm in AiZynthFinder does not include a filter to quickly remove unfeasible reactions nor does it utilize different policies for the expansion and rollout phases.

**Fig. 1:**
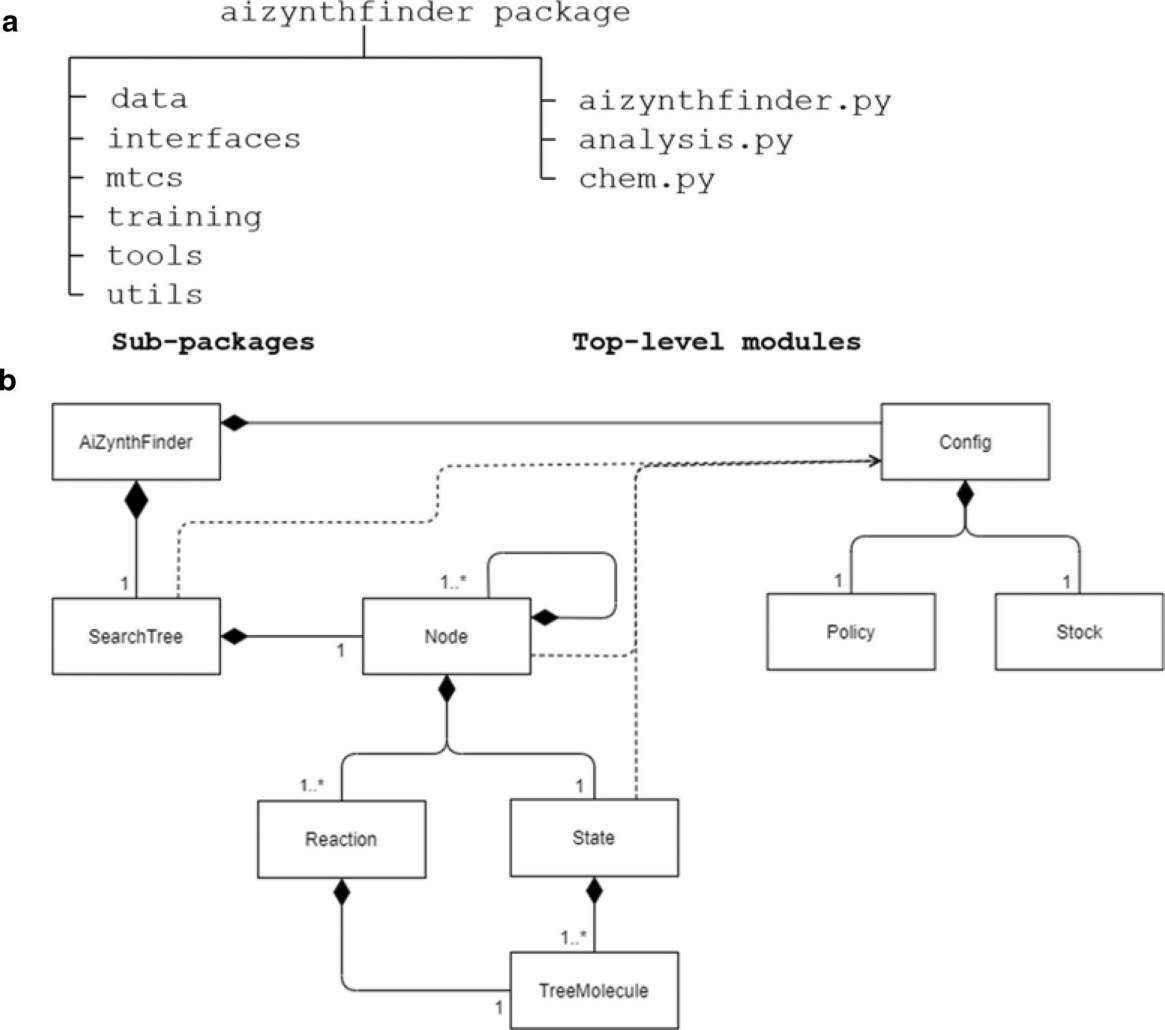
The AiZynthFinder package. **a** The python package structure, outlining top-level modules and sub-packages. **b** The main classes involved in the tree search and the relationships. A line ending with a solid diamond indicates an “owns”-relation, and a line ending with arrow indicates an “uses”-relationship, according to UML notation

The structure of the AiZynthFinder package is shown in Fig. 1a. The main interface to the algorithm is in the aizynthfinder.py module, which brings classes from the mcts sub-package together to perform the tree search. However, for the end-user we provide two interfaces: one command-line interface (CLI) and one graphical user interface (GUI) that is intended to be used in a Jupyter notebook. These two interfaces, which reside in the interface sub-package, are installed together with the package. The CLI comes with some additional features that are lacking from the GUI. Foremost, it allows compounds to be processed in batch, i.e. the user can submit hundreds or thousands of compounds with one command. Secondly, detailed results are stored to disc that later can be processed or viewed. For instance, one can calculate statistics on the search trees, or one can produce images of the top-ranked routes. Lastly, the CLI allows a finer detail of debugging information, which could be invaluable to software developers. The sub-package training contains tools to train the policy neural network, and the sub-package tools contains other useful CLIs.

The overall design follows principles from object-oriented programming such that each component is implemented as a class. The main classes for the tree search and their relationships are shown in Fig. 1b. The AiZynthFinder class loads a user configuration from file as a Configuration object, which includes the creation of a Policy and a Stock object. This configuration is used to control the tree search. The actual tree search is then carried out by the TreeSearch class that creates a Node object representing a node in the tree search that can be expanded to create new Nodes. The molecules on each Node are represented by a State object that holds a list of TreeMolecule objects. A Reaction class encapsulates a chemical reaction on TreeMolecule objects and is used to apply the reaction templates to create new precursors.

The Policy class encapsulates a recommendation engine based on a trained neural network. Given a molecule object, it will return a sorted list of reaction templates and the probability of each template. The templates are sorted on the probability as given by the neural network. We have trained neural networks on several template libraries (see ref [24] for a comparison) and provide one based on the publicly available US patent office data (USPTO) set [30] for anyone to use. We also provide tools to train the neural network, in case someone has their own or in-licensed reaction database. These tools can for instance be used with RDChiral [31] and our previously described procedure [24] for extracting templates.

The Stock class is an abstraction around a collection of compounds that serves as stop-conditions for the tree search. This is a list of purchasable compounds, but could also be an abstract collection based on some rules, e.g. compounds with less than seven carbon atoms are considered purchasable. To support different kinds of collections, the Stock class uses one or more instances of query classes that given a molecule object returns whether that compound is “in stock”. The package comes with two query classes, one that holds a set of InChI keys [32] in the computer memory and one that holds a connection to a Mongo database with InChI keys. We also provide examples to show how one can create a rule-based query class. For our internal usage we refer to lists of purchasable compounds from several commercial vendors, however it is just as straightforward to create a list from open source databases such as ZINC [33]. To simplify this process, we provide a tool to make a stock in a suitable format for the tree search from files containing SMILES strings [34].

The main MCTS implementation has been extensively profiled and optimized—the bottlenecks are calls to the neural network and to RDChiral [31] for resolving reaction templates, routines that rely on optimized C or C++ code. We have not attempted to parallelize the code, as the serial execution time is sufficient for our purposes (see below). For the prediction of multiple compounds at the same time, the code can of course be embarrassingly parallelized. The benchmarking numbers below were made using a single CPU (Intel Xeon 4.00 GHz) and a single GPU (Nvidia GeForce RTX 2080 Ti) on a Linux machine with 64 GB memory.

More than 85% of the code is covered by automatic unit and integration tests, which we execute on each commit. Furthermore, the code is pep8 compliant, autoformatted and code complexity is monitored automatically on each commit. All of this contributes to the robustness and maintainability of the code base and provides the basis for continuous integration and deployment. Extensive API documentation is autogenerated from docstrings and is complemented by hand-written tutorials.

## Results and discussion

As described in the Implementation section, there are two main interfaces to the tool. Here, we exemplify the usages of the tool with the GUI and then proceed with a comparison using the CLI. In the example below we have used the policy trained on USPTO data [24]. Furthermore, we created a stock from compounds available in the ZINC database [33]; we only downloaded tranches including fragment compounds (molecular weight up to 250 D and log P up to 3.5) that had reactivity labeled as “standard” or “reactive”, resulting in 17,422,831 compounds.

## Graphical user interface

To use the GUI (and the CLI), a configuration file needs to be created in YAML-format. This configuration file must contain the path to files for the policy and instructions how to setup the stock. The policy files are (1) the saved neural network model and, (2) a list of reaction templates. Multiple stocks and policy networks can be specified in the configuration and selected in the GUI before running the algorithm. The user is also free to fine tune the search algorithm using a set of properties. For the GUI, they serve as default values whereas for the CLI they are used in the search algorithm. If not provided in the configuration file, default recommended settings are automatically applied.

The GUI is based on the Jupyter notebook infrastructure, which builds and displays the GUI requiring at minimum two lines of python code. Although, a Jupyter notebook requires the user to enter Python code, the number of commands one must enter is minimal so that it is suitable even for non-technical researchers. A Jupyter notebook is also ideal as a working environment for researchers that want to experiment with the algorithm and the result of the tree search. Because a Jupyter notebook provides the full Python environment, one can easily customize the setup of the algorithm and fully inspect the predicted routes. Furthermore, there are projects such as voilá [35] built around Jupyter notebooks that make it easy to create interactive webpages directly from the notebooks. This could be setup for users that primarily want to use AiZynthFinder to find suggestions for synthesis plans.

**Fig. 2:**
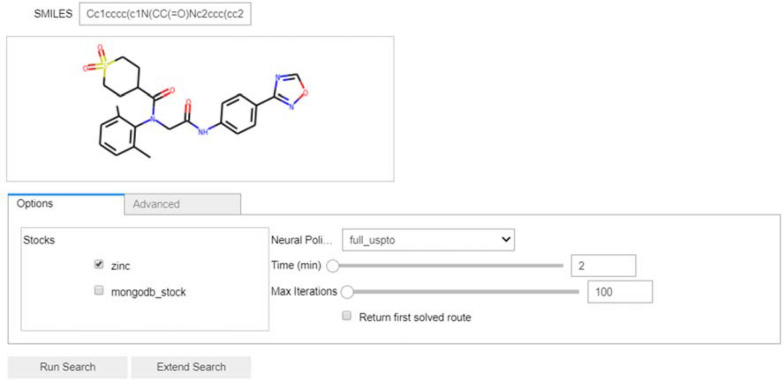
The input section of the AiZynthFinder GUI. A user has entered the SMILES string for the drug Amenamevir and selected the ZINC stock

In Fig. 2, we have input the SMILES string for the antiviral drug Amenamevir. Furthermore, the user can then select the stock and neural policy they want to use, as well as some options for the tree search.

**Fig. 3:**
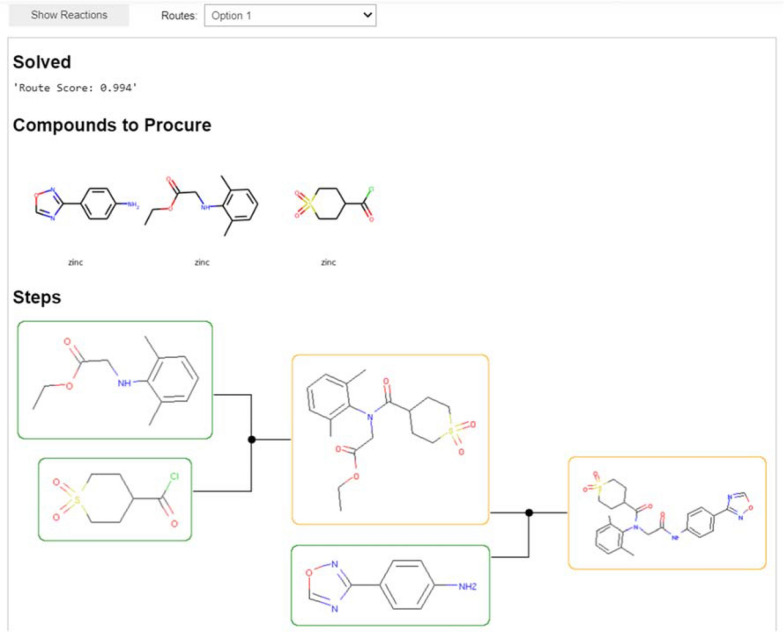
The output section of the AiZynthFinder GUI displaying the first suggested route to synthesize Amenamevir

When the tree search is completed, the user can view the predicted reaction routes. The GUI allows browsing through the top-ranked routes, but using Python scripting, all routes can be extracted and displayed. Figure 3 shows an example for the Amenamevir drug. First, the results show whether the route is solved or not, i.e. if all precursors are in stock, and the score of the route. The score reflects the fraction of solved precursors and the number of reactions required to synthesize the target compound. The score for a solved compound is close to 1.0, whereas the score for an unsolved compound is typically less than 0.8. However, it should be noted that the score was designed to support the tree search and is rather indiscriminate with regard to the quality of the route (i.e. if it’s a good route or not) and should be interpreted with care. Second, the results clearly display which precursors to procure in order to synthesize the target compound. Lastly, it shows the predicted route with precursors in stock highlighted with a green rectangle, and the precursors that are not in stock are highlighted in orange. In the example shown in Fig. 3, we see that suggested route is very similar to the reported synthetic route for Amenamevir [36], with the difference that the anilinoacetate is available to purchase and does not need to be synthesized.

## Comparison with the ASKCOS tool

As mentioned above, several other retrosynthesis tools exist, but unfortunately very few of them are open source or well described in the literature. The software that is closest for a comparison is the Tree builder module in the ASKCOS suite of programs [14, 37]. First the algorithm underlying the Tree builder module is similar to the algorithm of AiZynthFinder, although different expansion policies are used, and the search tree constructed differently. The software is written in Python and the code is available on Github. However, it is foremost intended for end-users and the interface is web-based. LillyMol [15], which is another open-source code, uses an exhaustive search of template space to produce one-step suggestions, i.e. not complete routes, and is thus less relevant to compare with. To make a rough baseline comparison between ASKCOS and AiZynthFinder we selected 100 random compounds from the ChEMBL database and submitted them to the Tree builder module of the public ASKCOS web server [38]. Even though this might not represent the latest version of the codebase, it is intuitively the interface that most people would use. We set a max depth of 6, an expansion time of 120 s and used a fast filter; otherwise default values were applied. We used the AiZynthFinder CLI together with the ZINC stock and the USPTO policy to predict routes for the same 100 compounds. Some statistics on the source code and the route finding are collected in Table 1 and the full data is available as Additional file 1. It is important to note that these 100 compounds are not necessarily a representative part of the chemical space that might be relevant in a drug design project. Thus, the test set should be viewed as an illustration of the capacity of the software rather than a go-to benchmarking set.

**Table 1:** Statistics of AiZynthFinder and ASKCOS predictions on 100 compounds from ChEMBL

	AiZynthFinder	ASKCOS
Number of core statements^a^	1095	2336
Number of total statements^b^	1495	9987
Average code complexity^c^	2.2	3.4
Average code effort^d^	22.0	116.8
Reaction database	USPTO [30]	Reaxys [39]
Stock	ZINC [33]	Sigma and eMolecules [6]
Average search time^e^ (s)	38.7	151.0
Average solution time^f^ (s)	7.1	14.3
Number of solved routes	55	62
Average number of steps	2.4	3.3
Average number of precursors	2.7	3.2

^a^The number of Python statements in the modules that are used by the AiZynthFinder CLI and tree builder module, respectively

^b^The total number of python statements in the *aizynthfinder* and *makeit* (ASKCOS) python packages, respectively

^c^The average cyclomatic complexity over all functions used by the AiZynthFinder CLI or the tree builder module

^d^The average Halstead effort over all functions used by the AiZynthFinder CLI or the tree builder module

^e^The average time to complete the search over all compounds

^f^The average time to find the first solution over all compounds that were solved

**Fig. 4:**
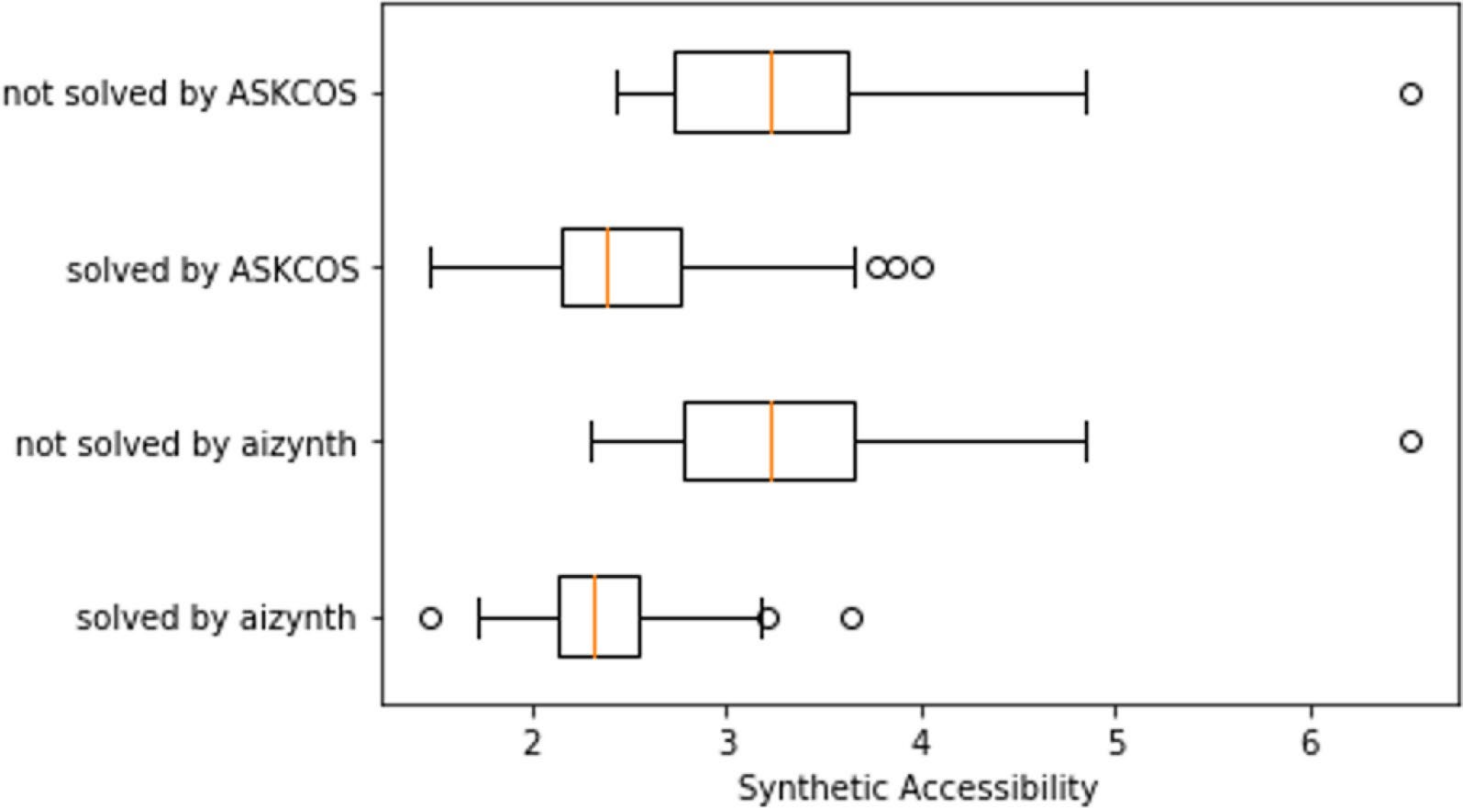
Distribution of the synthetic accessibility score of the 100 ChEMBL compounds, grouped by whether a synthetic route was found with AiZynthFinder or ASKCOS

AiZynthFinder and ASKCOS find routes for 55 and 62 compounds respectively. There were 47 compounds for which both tools found a route, 15 compounds where ASKCOS found a solution and AiZynthFinder did not, and 8 compounds where AiZynthFinder found a solution and ASKCOS did not. There were 30 compounds that neither tool found a solution for. We have found that route finding capability depends on the stock that is used as stop criteria in both tools [24]. The example stock created from a subset of the ZINC database is for instance much less extensive than some of the commercial stocks we typically use. If we include the readily available Enamine building blocks in the stock, we could find routes for an additional 10 compounds. The ASKCOS tool from the public webserver employs a commercial database consisting of 107,000 compounds with list prices less than $100/g from Sigma Aldrich and eMolecules [6]. The other factor that determines if a solution is found is the template library—here we used USPTO policy for AiZynthFinder, whereas ASKCOS is based on the more extensive Reaxys database [39]. Using a policy based on Reaxys data we find routes for 56 compounds, although there is not a complete overlap with the USPTO results. We have previously investigated the effect of policies trained on a variety of datasets on the route finding capability of AiZynthFinder [24] however we cannot release these to the public due to licensing agreements. Furthermore, the capability to find a route for both tools is closely related to the complexity of the synthesis. This can be seen in Fig. 4, showing the distribution of the synthetic accessibility (SA) score [40] for four sets of data. We see that for both AiZynthFinder and ASKCOS, the SA score is generally lower for compounds that the tools were able to find a solution for. Similar observations have been discussed previously in the literature [41]. It seems that ASKCOS is somewhat better at finding solutions with a mid-range SA score, but this might be due to the lack of some scaffolds in the ZINC stock. Moreover, it seems that AiZynthFinder predicts slightly shorter reaction routes, with fewer purchasable precursors, although it is unclear if the difference is significant given the rather small test set.

Looking at the timings of the software, we see that AiZynthFinder is faster than ASKCOS, both in terms of total search time and the time it takes to find the first solution. However this difference could be partially attributed to the environment in which the test was executed, a local Linux computer in the case of AiZynthFinder and a webserver in the case of ASKCOS. Lastly, we want to point out that AiZynthFinder has a much smaller code base than ASKCOS, with less than half the number of Python statements in the core modules (the part of the code necessary to execute the tree search). The large difference in total statements of the package can be attributed to the fact that ASKCOS has a lot more features than AiZynthFinder. However, the difference in the number of core statement could be because we re-engineered the AiZynthFinder package such that it is a better designed package than the previously released code. We quantify this by calculating the average complexity [42], which quantifies the number of independent branching points, and Halstead effort [43], which is the product of a volumetric measure and the difficulty to understand the code. The number of lines, the code complexity and code effort is among the metrics typically used to determine if a codebase is maintainable [44], and they indicate that the AiZynthFinder code is less complex and require less effort to extend than ASKCOS.

This is far from a comprehensive comparison and is intended to highlight the similarities and differences between the two tools. As mentioned above, it is difficult to compare the software on equal footing. Different researchers have different priorities when it comes to retrosynthesis, and it is not entirely clear how to make a good comparison. We have not discussed the quality of the predicted routes, which is in our opinion is an ill-defined metric. For instance, we submitted Amenamevir to the ASKCOS webserver and did not recover the expected literature route, but that does not mean that the route suggested by ASKCOS is incorrect. The only fair way to find out is to synthesize the compounds according to the proposed the route, but even then the successful application of the suggested route is conditioned on finding the optimal conditions for synthesis. As such, a comprehensive comparison of tools is out of scope for this software note.

## Future developments

It is our aim that the AiZynthFinder software provides a framework for research and development of novel retrosynthesis algorithms. Therefore, we have designed the software to be easy to maintain and extend with new features. Currently, it contains a solid foundation, i.e., the Monte Carlo tree search algorithm that has shown promising results in finding routes for a range of compounds. And we provide interfaces that suits this core activity. However, it does not yet provide a fully integrated solution. For instance, we are working on improving the accuracy of the predicted routes by implementing a scoring framework. It is also of interest to augment the predictions with an information retrieval system for the used templates, so that chemists can e.g. look up similar reactions. Finally, we are working on improving the recommendation policy, by for instance utilizing the “ring breaker” policy [45]. All such extensions should be possible to implement easily in the current codebase because it has low complexity and Halstead effort. If the features do not depend on internal AstraZeneca infrastructure or data, and are relevant to the larger community, they will be made available when we publish new research findings. We expect minor releases with new features to happen several times a year, whereas patch releases fixing bugs and trivial code updates will be released continuously.

## Conclusions

We have presented the AiZynthFinder tool for retrosynthesis planning. In our experience, it can suggest synthetic routes for most compounds in a very short time. We hope that it will be perceived as user-friendly and with a low learning curve, because we provide extensive documentation. Furthermore, the software is robust and flexible and lends itself to easy extension with novel features. Although it does not provide a complete and integrated solution for synthesis planning, we believe that we have provided a framework and platform where novel algorithms can be tested and integrated in the future. We hope that by releasing the software to the public that researchers interested in retrosynthesis can use it to explore synthetic route prediction and provide suggestion how it can be improved. By providing open source code and algorithmic transparency, we aim to promote collaboration around a sustainable reference software. We encourage users to contribute ideas or code so that the tool can be incrementally improved and thereby provide more accurate and useful predictions of reaction routes.

## Supplementary information

**Additional file 1.** Complete search results for comparison between AiZynthFinder and ASKCOS.

## Data Availability

Project name: AiZynthFinder Project home page: http://www.github.com/MolecularAI/aizynthfinder Operating system(s): Platform independent Programming language: Python 3 Other requirements: several open source python packages License: MIT. Any restrictions to use by non-academics: none.

## Data availability

The ZINC stock as well as the trained USPTO policy is available to download from Figshare: 10.6084/m9.figshare.12334577.v1.
