# Supplementary material for: AiZynthFinder: a fast, robust and flexible open-source software for retrosynthetic planning
Source: J Cheminform. 2020 Nov 17;12:70. doi: 10.1186/s13321-020-00472-1 (PMC7672904; doi:10.1186/s13321-020-00472-1)
Supplement: Supplementary file 1 — Additional file 1. Complete search results for comparison between AiZynthFinder and ASKCOS. [file 13321_2020_472_MOESM1_ESM.docx]

# Supporting information:

# AiZynthFinder: a fast, robust and flexible software for retrosynthetic planning

Samuel Genheden, Amol Thakkar, Veronika Chadimová, Jean-Louis Reymond, Ola Engkvist, Esben Bjerrum

**Table 1 - Full results of the 100 ChEMBL compounds submitted to AiZynthFinder and ASKCOS**

|  |  |  | AiZynthFinder | | | | | | ASKCOS | | | | |
| --- | --- | --- | --- | --- | --- | --- | --- | --- | --- | --- | --- | --- | --- |
| SMILES | **SA** | **Search time** | **Solution time** | **Solved** | **Solved with Reaxys^a^** | **Solved with BB^b^** | **Number of steps** | **Number of precursors** | **Search time** | **Solution time** | **Solved** | **Number of steps** | **Number of precursors** |
| **O=C(NCc1cccc(Cl)c1)c1ccc2c(c1)OCO2** | 1.7 | 4.6 | 0.0 | Yes | Yes | Yes | 1 | 2 | 121.7 | 10.1 | Yes | 1 | 2 |
| **CCc1cc(=Nc2ccc3ccccc3c2)n2[nH]cnc2n1** | 2.9 | 41.2 |  | No | No | No |  |  | 10.2 |  | No |  |  |
| **CC(=O)C1=C(C)N=C2CC(C)(C)CC(=O)C2C1C** | 3.7 | 58.6 |  | No | No | No |  |  | 120.9 |  | No |  |  |
| **O=C(Nc1scc(-c2cccs2)c1C(=O)O)c1cccs1** | 2.3 | 21.3 | 0.4 | Yes | Yes | Yes | 1 | 2 | 123.7 | 10.1 | Yes | 2 | 2 |
| **O=C1NCN(c2ccccc2)C12CCN(C(c1cccs1)**  **c1nnnn1Cc1ccccc1)CC2** | 3.6 | 53.5 |  | No | Yes | No |  |  | 122.8 |  | No |  |  |
| **Cc1ocnc1C(=O)Nc1ccc(N2C(=O)c3cccnc3C2=O)c(Cl)c1** | 2.5 | 36.8 | 13.3 | Yes | Yes | Yes | 2 | 3 | 126.3 | 10.1 | Yes | 3 | 3 |
| **O=C(c1cnc(-c2ccc(Cl)cc2)s1)c1ccc(Cl)cc1Cl** | 2.1 | 13.3 | 0.0 | Yes | Yes | Yes | 1 | 2 | 152.6 | 10.1 | Yes | 2 | 3 |
| **CCCCn1c(=S)[nH]c2ccc(N3CCOCC3)cc2c1=O** | 2.3 | 15.9 | 0.0 | Yes | Yes | Yes | 1 | 2 | 123.1 | 45.2 | Yes | 5 | 4 |
| **CCC(=O)N(c1ccccc1)C1CCN(C(=O)C**  **(Cc2ccccc2)NC(=O)C2Cc3ccccc3CN2C(=O)C(N)Cc2c(C)**  **cc(O)cc2C)CC1** | 4.0 | 81.0 |  | No | No | Yes |  |  | 270.1 | 16.1 | Yes | 7 | 5 |
| **COc1cccc(CNC(=O)CC(NC(C)=O)c2ccccc2)c1** | 2.2 | 33.3 | 28.1 | Yes | Yes | Yes | 3 | 3 | 132.3 | 10.1 | Yes | 1 | 2 |
| **Cn1cnc(S(=O)(=O)N(Cc2ccsc2)C2Cc3cc(C#N)ccc3N**  **(Cc3cncn3C)C2)c1** | 3.7 | 67.5 |  | No | No | No |  |  | 123.3 |  | No |  |  |
| **Cc1cccc2cc(Cn3nc(C#CC(C)O)c4c(=N)[nH]cnc43)n**  **(-c3ccccc3Cl)c(=O)c12** | 3.8 | 78.3 |  | No | Yes | Yes |  |  | 121.6 |  | No |  |  |
| **Nc1c(SCCC(=O)O)ccc2c1C(=O)c1ccccc1C2=O** | 2.3 | 34.5 | 14.3 | Yes | Yes | Yes | 3 | 2 | 147.8 | 10.1 | Yes | 1 | 2 |
| **COc1ccc(CC2=NC(=S)N(CN3CCOCC3)C2=O)cc1** | 2.6 | 57.7 |  | No | No | No |  |  | 121.5 |  | No |  |  |
| **CC(=NOC(=O)C1CC1)C1CC1c1ccc(F)cc1** | 3.2 | 30.9 | 14.9 | Yes | Yes | Yes | 3 | 3 | 132.0 | 10.1 | Yes | 7 | 4 |
| **O=C(COC(=O)c1cccnc1Cl)c1ccc(OC(F)F)cc1** | 2.0 | 21.7 | 0.0 | Yes | Yes | Yes | 2 | 2 | 126.9 | 10.1 | Yes | 1 | 2 |
| **Cc1ccc(-c2c(-c3ccccc3)sc(N)c2C(=O)c2ccc(Cl)cc2)cc1** | 2.1 | 33.4 | 0.0 | Yes | No | Yes | 4 | 5 | 132.1 | 10.1 | Yes | 5 | 3 |
| **Cc1[nH]n(-c2ccccc2)c(=S)c1C=Nc1ccc([N+]**  **(=O)[O-])cc1[N+](=O)[O-]** | 2.9 | 50.7 |  | No | Yes | Yes |  |  | 10.1 |  | No |  |  |
| **COc1cc(OC)c2sc(-c3ccc(-n4cnnc4-c4cc**  **(OC)c(OC)c(OC)c4)cc3)nc2c1** | 2.7 | 56.0 |  | No | Yes | Yes |  |  | 121.3 |  | No |  |  |
| **CC(=O)Nc1c(C)c(=O)n(C)c2nc(-c3ccc(Cl)**  **cc3Cl)c(-c3ccc(Cl)cc3)cc12** | 2.5 | 44.2 |  | No | No | No |  |  | 121.4 |  | No |  |  |
| **CN1C(=O)c2ccc(C(=O)N=c3ccc(Br)c[nH]3)cc2C1=O** | 2.9 | 57.3 |  | No | No | No |  |  | 121.4 |  | No |  |  |
| **CC(=NNC(N)=O)c1ccc(Cl)cc1** | 1.9 | 15.7 | 2.3 | Yes | Yes | Yes | 1 | 1 | 10.1 | 10.1 | Yes | 1 | 1 |
| **O=C1c2ccccc2C(=O)N1CC(CN1C(=O)c2ccccc2C1=O)**  **(C(=O)OCc1ccccc1)C(=O)OCc1ccccc1** | 2.6 | 29.3 | 9.7 | Yes | Yes | Yes | 4 | 5 | 120.9 |  | No |  |  |
| **Cc1ccc2c(c1)CCCN2C(=O)CCc1nc(=O)c2ccccc2[nH]1** | 2.2 | 24.3 | 0.0 | Yes | Yes | Yes | 1 | 2 | 128.7 | 10.1 | Yes | 1 | 2 |
| **Cn1ccc2ccc3c(c21)CCN3C(=O)Nc1cccnc1** | 2.5 | 32.7 | 9.3 | Yes | Yes | Yes | 6 | 3 | 121.4 |  | No |  |  |
| **COC12C(=COC(N)=O)c3c(O)c(N)c(C)c(O)**  **c3N1CC1C2N1C(=S)Nc1ccccc1** | 4.9 | 51.0 |  | No | Yes | No |  |  | 121.2 |  | No |  |  |
| **O=C1NC(=S)SC1=Cc1ccc(O)c(Br)c1** | 2.6 | 8.9 | 0.0 | Yes | Yes | Yes | 1 | 2 | 10.1 | 10.1 | Yes | 1 | 2 |
| **CCCCCOC(=O)N1CCN(C(=O)C(CCC(=O)O)NC(=O)**  **c2cc(N3CCC(COC)C3)cc(-c3ccccc3)n2)CC1** | 3.5 | 54.0 |  | No | No | No |  |  | 292.0 | 209.5 | Yes | 11 | 6 |
| **Cc1cc(N=c2nc3c(c[nH]2)CCc2nn(C)c(-c4ccc**  **(OC(F)F)cc4)c2-3)nn1C** | 3.6 | 62.5 |  | No | No | No |  |  | 121.3 |  | No |  |  |
| **O=C(Nc1ccc(F)cc1F)C1(c2cccs2)CCOCC1** | 2.4 | 15.2 | 0.0 | Yes | Yes | Yes | 1 | 2 | 292.0 | 10.1 | Yes | 5 | 5 |
| **O=C(CSc1nnc(C2CC2)n1C1CC1)c1cccs1** | 2.4 | 17.6 | 0.5 | Yes | Yes | Yes | 1 | 2 | 122.1 | 10.1 | Yes | 3 | 3 |
| **COc1ccc(-n2c(SCC(=O)NCC3CCCO3)**  **nc3ccccc3c2=O)cc1Cl** | 2.7 | 68.5 | 59.1 | Yes | No | Yes | 3 | 3 | 124.4 | 10.1 | Yes | 2 | 3 |
| **O=C(CC[N+](=O)[O-])N=c1ccc(Cl)c[nH]1** | 3.6 | 31.5 | 0.0 | Yes | Yes | Yes | 1 | 2 | 122.0 |  | No |  |  |
| **CC=Cc1ccc2c(=NC(C)c3ccc(NC(=O)c4ccc(Cl)**  **nc4)cc3)[nH]c(N(C)C)nc2c1** | 3.5 | 87.2 |  | No | No | No |  |  | 121.3 |  | No |  |  |
| **Brc1ccc(C2=CSC3=NC4=C(CNCC4=Cc4ccccc4)**  **C(c4ccccc4)N23)cc1** | 3.4 | 56.9 |  | No | No | No |  |  | 120.9 |  | No |  |  |
| **O=c1c2c3c(sc2ncn1-c1ccccc1)CCCC3** | 2.1 | 19.7 | 0.4 | Yes | Yes | Yes | 1 | 2 | 123.1 | 10.1 | Yes | 1 | 2 |
| **C=CCN1C(=O)c2ccc(C(=O)NCC(=O)Nc3c(C)**  **cc(C)cc3C)cc2C1=O** | 2.3 | 11.1 | 0.0 | Yes | Yes | Yes | 2 | 3 | 135.6 | 10.1 | Yes | 2 | 3 |
| **COc1ccccc1NC(=S)N1CCC(c2nc3cc(C)c(C)**  **cc3[nH]2)CC1** | 2.3 | 4.8 | 0.0 | Yes | Yes | Yes | 1 | 2 | 208.4 | 10.1 | Yes | 3 | 5 |
| **Cc1nc2[nH]c(SCc3nc4ccccc4[nH]3)nc2cc1Br** | 2.7 | 16.0 | 0.0 | Yes | Yes | Yes | 1 | 2 | 126.5 | 10.1 | Yes | 2 | 3 |
| **COc1cc(O)c2c(c1)C(=O)c1cc(CN(CCO)CCO)**  **cc(OC)c1C2=O** | 2.6 | 42.8 | 44.1 | Yes | No | No | 6 | 3 | 121.9 |  | No |  |  |
| **CCC(=O)c1c(Cl)n(C2OC(COC(C)=O)C(O)C2O)**  **c2cc(Cl)c(Cl)cc12** | 3.9 | 38.9 |  | No | Yes | No |  |  | 121.3 |  | No |  |  |
| **COc1ccc(OCCNC(=O)c2c(C)nc3cc(C)ccn23)cc1** | 2.1 | 5.0 | 0.0 | Yes | Yes | Yes | 1 | 2 | 187.1 | 10.1 | Yes | 3 | 3 |
| **Cc1cc(OCC(O)CN2C(C)CCCC2C)ccc1C(C)C** | 3.2 | 51.1 | 34.5 | Yes | No | Yes | 3 | 3 | 209.6 | 10.1 | Yes | 2 | 3 |
| **COc1cc(N=Nc2ccc(S(=O)(=O)O)cc2)**  **ccc1N=Nc1ccc(O)c(C(=O)O)c1** | 2.8 | 62.2 |  | No | No | No |  |  | 198.1 | 10.1 | Yes | 1 | 3 |
| **N#Cc1ccc(NC(=O)NCc2cccc(C(=O)**  **Nc3ccc(CN)cc3)c2)cc1** | 2.0 | 28.0 | 0.0 | Yes | Yes | Yes | 3 | 3 | 237.1 | 10.1 | Yes | 4 | 3 |
| **CC(C)(C)CNC(=O)N(CCCl)N=O** | 3.0 | 35.5 | 0.3 | Yes | No | Yes | 2 | 2 | 151.6 | 10.1 | Yes | 2 | 2 |
| **O=C(CN1C(=O)C(=Cc2cccc(F)c2)Oc2ccccc21)**  **N1CCN(c2ccccn2)CC1** | 2.5 | 43.1 | 5.1 | Yes | Yes | Yes | 6 | 4 | 172.1 | 10.1 | Yes | 5 | 4 |
| **COc1cc(N)c(Cl)cc1C(=O)OCCN1CCN(C(=O)**  **CCCNS(=O)(=O)c2cccc3c(N(C)C)cccc23)CC1** | 2.8 | 63.4 |  | No | Yes | Yes |  |  | 247.8 | 10.1 | Yes | 7 | 5 |
| **NCCn1nc2c3c(c(NCCN4CCOCC4)ccc31)**  **C(=O)c1ccncc1-2** | 2.8 | 40.2 |  | No | No | No |  |  | 122.1 |  | No |  |  |
| **N#Cc1ccc(Cn2ccnc2)cc1Oc1ccc(Cl)cc1** | 2.1 | 17.4 | 0.0 | Yes | Yes | Yes | 1 | 2 | 213.5 | 10.1 | Yes | 3 | 4 |
| **Cc1ccc2ncc(S(=O)(=O)c3ccccc3)c(N3CCC(C)CC3)c2c1** | 2.2 | 39.0 | 0.0 | Yes | Yes | Yes | 5 | 5 | 127.6 | 10.1 | Yes | 4 | 3 |
| **CC(=O)N1CCN(CC(=O)N2CCN(c3cc4c(cc3F)**  **c(=O)c(C(=O)O)cn4C3CC3)CC2)CC1** | 2.6 | 40.4 | 0.0 | Yes | No | Yes | 4 | 5 | 235.7 | 10.1 | Yes | 2 | 3 |
| **c1ccc(-c2nc(=NC3CC3)c3ccccc3[nH]2)cc1** | 2.4 | 26.4 | 0.0 | Yes | Yes | Yes | 1 | 2 | 121.0 |  | No |  |  |
| **COc1cc(OCCCN(C)C)c2c(c1)C(C)(C)**  **C(c1ccccc1)C2c1ccccc1** | 3.3 | 60.3 |  | No | No | No |  |  | 121.6 |  | No |  |  |
| **CCN(CC(=O)Nc1ccccc1C(F)(F)F)C(=O)**  **c1ccc(-n2nc(C)c(CCC(=O)OC)c2C)cc1** | 2.5 | 49.0 | 37.3 | Yes | No | Yes | 6 | 4 | 243.0 | 17.1 | Yes | 6 | 5 |
| **Cn1cc(-c2ncc3c(c2C(=O)O)CCN(Cc2ccco2)C3)cn1** | 2.7 | 36.7 |  | No | Yes | No |  |  | 123.4 |  | No |  |  |
| **CCc1c2n(C)ccc3c-2n(c(=O)c1=O)c1ccccc31** | 2.9 | 56.1 |  | No | No | No |  |  | 121.2 |  | No |  |  |
| **CCCc1nc(C(=O)NC2COCC2N2CCN(C)CC2)cs1** | 3.4 | 43.2 |  | No | No | Yes |  |  | 122.9 |  | No |  |  |
| **Cn1cc(C2=CC3c4cccc(N5CCOCC5)**  **c4CCN3C(=O)C=N2)cn1** | 3.7 | 47.5 |  | No | No | No |  |  | 121.9 |  | No |  |  |
| **CCCCc1nn2ncccc2c1-c1cc[nH]**  **c(=Nc2ccc(N3CCN(C)CC3)cc2)n1** | 3.2 | 56.1 |  | No | No | No |  |  | 122.6 |  | No |  |  |
| **O=C(OCCN1CCCC1)c1ccccc1N=c1cc[nH]**  **c2cc(C(F)(F)F)ccc12** | 2.7 | 48.6 |  | No | No | No |  |  | 262.3 | 17.7 | Yes | 4 | 5 |
| **CN1C(=O)C(OC(=O)C(Cl)(Cl)Cl)N=C(c2ccccc2)**  **c2cc(Cl)ccc21** | 3.2 | 45.8 |  | No | No | No |  |  | 123.5 | 10.1 | Yes | 2 | 2 |
| **COc1ccccc1NS(=O)(=O)CC12CCC(CC1=O)C2(C)C** | 3.9 | 57.2 |  | No | Yes | No |  |  | 122.0 | 10.1 | Yes | 1 | 2 |
| **CCS(=O)(=O)c1ccc2[nH]c(O)c(C=c3[nH**  **]c4c(c3=CCCN3CCN(CCO)CC3)CCCC4)c2c1** | 3.4 | 45.6 |  | No | No | No |  |  | 121.6 |  | No |  |  |
| **O=C(C(=O)N1Cc2ccccc2C1)c1c[nH]c2ccccc12** | 2.1 | 12.9 | 0.0 | Yes | Yes | Yes | 1 | 2 | 184.0 | 10.1 | Yes | 1 | 3 |
| **O=C1CCc2cc(OCCCCN3CCN(c4cccc**  **(Cl)c4Cl)CC3)ccc2N1** | 2.3 | 29.3 | 0.0 | Yes | Yes | Yes | 2 | 3 | 265.4 | 10.1 | Yes | 2 | 3 |
| **O=C(C=Cc1ccc(O)c(O)c1)Nc1ccccc1O** | 2.0 | 16.4 | 0.0 | Yes | Yes | Yes | 1 | 2 | 222.7 | 10.1 | Yes | 1 | 2 |
| **CC(C)(C)NC(=O)N1CCN(C2c3ccc(Cl)**  **cc3CCc3cc(Br)cnc32)CC1C(=O)NCc1cccnc1** | 3.7 | 51.2 |  | No | No | Yes |  |  | 217.5 | 10.1 | Yes | 11 | 6 |
| **Cc1nn(C2CCOCC2)c2sc(C(=O)NC3CCC**  **(N4CCC(CO)CC4)CC3)cc12** | 2.8 | 52.1 | 0.0 | Yes | No | Yes | 4 | 4 | 123.7 |  | No |  |  |
| **COc1ccc2c(c1)c(C1C(C)C3CCCCC3CN1S**  **(=O)(=O)c1ccc(C)cc1)cn2C** | 3.7 | 63.4 |  | No | Yes | No |  |  | 122.0 |  | No |  |  |
| **Oc1ccc(-c2nsc(-c3ccc(O)c(O)c3)n2)cc1O** | 2.5 | 31.9 | 0.0 | Yes | Yes | Yes | 2 | 3 | 259.2 | 10.1 | Yes | 3 | 2 |
| **Cc1c(CC(=O)O)c2cc(F)ccc2n1Cc1nc2ccccc2s1** | 2.3 | 31.6 | 0.6 | Yes | Yes | Yes | 1 | 2 | 145.3 | 10.1 | Yes | 4 | 4 |
| **CC(C)C(=O)NC(c1ccc(C(F)(F)F)cc1)c1cnccn1** | 2.8 | 28.1 | 25.1 | Yes | Yes | Yes | 2 | 3 | 228.4 | 10.1 | Yes | 4 | 4 |
| **Cc1cccnc1CN1CCN(Cc2ccc(-c3cc[nH]n3)o2)CC1** | 2.7 | 21.1 | 0.0 | Yes | Yes | Yes | 1 | 2 | 123.3 |  | No |  |  |
| **COc1cc(SC)ccc1C(=O)N(C)CC(=O)Nc1cccc2ccccc12** | 2.1 | 20.0 | 0.0 | Yes | Yes | Yes | 1 | 2 | 202.5 | 14.5 | Yes | 4 | 5 |
| **Cc1ccccc1C=CC(=O)NC(C)CO** | 2.5 | 40.6 |  | No | No | Yes |  |  | 202.4 | 10.1 | Yes | 1 | 2 |
| **COc1ccc(Oc2ccc(-c3cc4ccc(C(=N)N)cc4[nH]3)cc2)cc1** | 2.1 | 37.5 | 0.0 | Yes | Yes | Yes | 2 | 3 | 191.7 | 10.1 | Yes | 6 | 3 |
| **CC(=O)N1CCN(Cc2ccc3[nH]c(-c4cc5cc(-c6cn[nH]c6)**  **ccc5[nH]c4=O)cc3c2)CC1** | 2.7 | 40.0 | 31.9 | Yes | Yes | Yes | 7 | 6 | 124.1 |  | No |  |  |
| **COc1ccc2[nH]cc(CCNC(=O)c3cc(=O)c4c(OCc5ccccc5)**  **cccc4o3)c2c1** | 2.4 | 23.0 | 0.0 | Yes | Yes | Yes | 2 | 3 | 240.0 | 10.1 | Yes | 3 | 4 |
| **Cc1ccc(S(=O)(=O)N(C)CC2Oc3c(NC(=O)c4ccncc4)**  **cccc3C(=O)N(C(C)CO)CC2C)cc1** | 3.5 | 51.7 |  | No | No | No |  |  | 121.7 |  | No |  |  |
| **O=C(O)C1CCN(c2ccc(S(=O)(=O)N3CCC**  **(C(=O)O)CC3)cc2[N+](=O)[O-])CC1** | 2.3 | 43.0 |  | No | Yes | Yes |  |  | 192.3 | 10.1 | Yes | 1 | 2 |
| **Cc1nc(C)n(CC2CCCN2CC(=O)NCc2ccco2)n1** | 3.0 | 36.2 | 24.3 | Yes | No | Yes | 4 | 3 | 169.1 | 10.1 | Yes | 3 | 3 |
| **O=C(NC1CC1c1ccccc1)c1ccc([N+](=O)[O-])cc1** | 2.5 | 38.0 | 8.3 | Yes | Yes | Yes | 4 | 4 | 128.6 | 10.1 | Yes | 1 | 2 |
| **CCN(CC)c1ccc(-c2oc3ccccc3c(=O)c2O)cc1** | 2.1 | 39.5 | 0.6 | Yes | Yes | Yes | 1 | 2 | 163.3 | 10.1 | Yes | 2 | 2 |
| **O=C(O)c1cccc(CNCc2ccccc2)c1** | 1.5 | 11.3 | 0.0 | Yes | Yes | Yes | 1 | 2 | 174.2 | 10.1 | Yes | 3 | 3 |
| **CC1=NC(=O)NC(c2cc(Br)c(O)c(Br)c2)C1C**  **(=O)c1ccccc1** | 3.4 | 46.1 |  | No | No | No |  |  | 122.1 |  | No |  |  |
| **O=C(C(=O)N1CCN(C(=O)c2ccccc2)CC1)**  **c1c[nH]c2c(Cl)cccc12** | 2.2 | 22.5 | 0.5 | Yes | Yes | Yes | 1 | 2 | 134.3 | 10.1 | Yes | 1 | 3 |
| **COc1ccc(CN(Cc2nc(=O)c3ccccc3[nH]2)**  **C(=O)NC2CCCCC2)cc1** | 2.2 | 20.3 | 0.0 | Yes | Yes | Yes | 2 | 3 | 136.9 | 10.1 | Yes | 2 | 4 |
| **O=C(O)CN(c1ccccn1)S(=O)(=O)c1ccccc1** | 2.0 | 28.4 | 0.0 | Yes | Yes | Yes | 1 | 2 | 131.5 | 10.1 | Yes | 3 | 3 |
| **CCCOc1ccc(C2C(=O)N(C3CCCCCC3)**  **CC(=O)N2C(C)C)cc1OC** | 3.0 | 34.2 |  | No | No | No |  |  | 164.1 | 11.7 | Yes | 7 | 5 |
| **CCC(Cc1ccc(C(=O)NCc2ccccc2Br)cc1)C(=O)O** | 2.3 | 38.3 | 22.9 | Yes | No | Yes | 3 | 3 | 221.5 | 10.1 | Yes | 3 | 3 |
| **OCC1CC(n2cnc3c(SCc4ccc(Br)cc4)ncnc32)**  **C(O)C1O** | 3.7 | 38.5 |  | No | No | No |  |  | 122.7 |  | No |  |  |
| **O=c1c2ccccc2oc2c([N+](=O)[O-])ccc**  **(Cn3ccnc3)c12** | 2.6 | 31.1 | 4.8 | Yes | No | Yes | 3 | 2 | 164.6 | 10.1 | Yes | 3 | 3 |
| **O=C1CC(c2ccccc2)c2c(sc(-c3ccc(Cl)cc3)**  **c2-c2ccc(Cl)cc2)N1** | 2.9 | 38.7 |  | No | No | No |  |  | 123.3 | 10.1 | Yes | 3 | 4 |
| **CCCC(c1nnnn1Cc1ccco1)N1CCN(c2ccccc2)CC1** | 2.8 | 36.9 |  | No | No | Yes |  |  | 262.9 | 10.1 | Yes | 9 | 5 |
| **COc1ccc(C2=C(c3ccc(OC)c(OC)c3)**  **CN3CCCC3C2)cc1OC** | 2.8 | 38.8 |  | No | No | No |  |  | 128.8 | 10.1 | Yes | 7 | 5 |
| **COC12CCC3(CC1C(C)(O)C1CCC1)**  **C1Cc4ccc(O)c5c4C3(CCN1CC1CCC1)C2O5** | 6.5 | 46.1 |  | No | No | No |  |  | 121.5 |  | No |  |  |
| **COc1ccc(C2C(CCCCc3ccccc3)C(=O)**  **N2c2ccc(OC)cc2)cc1** | 2.7 | 48.9 |  | No | No | No |  |  | 121.6 |  | No |  |  |
| **Cc1cccc(C)c1NC(=O)C1=C(C(=O)O)C2CCC1C2** | 3.8 | 48.3 |  | No | Yes | No |  |  | 144.4 | 10.1 | Yes | 2 | 2 |
| **Cc1cc(C)n(-c2nc3ccccc3[nH]c2=Nc2cc(C(=O)O)ccc2C)n1** | 2.7 | 56.3 |  | No | No | No |  |  | 123.2 |  | No |  |  |

^a^ Solution found if expansion policy based on Reaxys data was used. ^b^ Solution found if the enamine building blocks where added to the ZINC stock.
